# Glutamatergic and GABAergic Metabolism in Mouse Brain under Chronic Nicotine Exposure: Implications for Addiction

**DOI:** 10.1371/journal.pone.0041824

**Published:** 2012-07-25

**Authors:** Mohammad Shameem, Anant Bahadur Patel

**Affiliations:** NMR Microimaging and Spectroscopy, Centre for Cellular and Molecular Biology (CCMB), Council of Scientific and Industrial Research (CSIR), Hyderabad, India; Weizmann Institute of Science, Israel

## Abstract

**Background and Purpose:**

The effects of nicotine on cerebral metabolism and its influence on smoking behavior is poorly understood. An understanding of the chronic effects of nicotine on excitatory and inhibitory metabolic demand, and corresponding neurotransmission may provide clues for designing strategies for the optimal smoking cessation intervention. The objective of the current study was to investigate neuronal and astroglial metabolism in mice exposed to nicotine (0.5 and 2.0 mg/kg, sc) three times in a day for 4 weeks.

**Experimental Approach/Principal Findings:**

Metabolic measurements were carried out by co-infusing [U-^13^C_6_]glucose and [2-^13^C]acetate, and monitoring ^13^C labeling of amino acids in brain tissue extract using ^1^H-[^13^C] and ^13^C-[^1^H]-NMR spectroscopy. Concentration of ^13^C-labeled glutamate-C4 was increased significantly from glucose and acetate with chronic nicotine treatment indicating an increase in glucose oxidation by glutamatergic neurons in all brain regions and glutamate-glutamine neurotransmitter cycle in cortical and subcortical regions. However, chronic nicotine treatment led to increased labeling of GABA-C2 from glucose only in the cortical region. Further, increased labeling of glutamine-C4 from [2-^13^C]acetate is suggestive of increased astroglial activity in subcortical and cerebellum regions of brain with chronic nicotine treatment.

**Conclusions and Significance:**

Chronic nicotine exposure enhanced excitatory activity in the majority of brain regions while inhibitory and astroglial functions were enhanced only in selected brain regions.

## Introduction

Nicotine addiction, a psychiatric disorder that underlies the widespread use of tobacco products, contributes to a large number of chronic illnesses and is a leading cause of deaths globally [1]. Although dysfunction of the brain reward circuitry involving the dopaminergic system has been implicated in nicotine addiction [2], impairment in cortical glutamatergic neurotransmission is also crucial for long term addictions [3]. Nicotine enhances the release of neurotransmitters glutamate, GABA, dopamine and serotonin by binding to nicotinic acetylcholine receptors (nAChRs) [4], [5], [6], [7]. Glutamate and GABA are the major excitatory and inhibitory neurotransmitters, respectively in the matured mammalian central nervous system which play major roles in glucose and energy metabolism, cortical excitability and cognitive function [8], [9], [10], [11]. ^13^C NMR studies have shown that the glutamate-glutamine cycle accounts for a major fraction of glutamine synthesis [12], and most importantly has a metabolic rate similar to neuronal glucose oxidation [13], [14], [15], [16]. Moreover, the rate of neurotransmitter cycle and neuronal mitochondrial glucose oxidation increased proportionately with a near 1∶1 slope [8], [16], [17], indicating that neurotransmitter energetics is supported by neuronal oxidative glucose metabolism. Alterations in glutamate and GABA pathways are associated with many neurological and neuropsychiatric disorders [18], [19], [20], [21].

Brain imaging studies in human have shown that nicotine increases the cerebral metabolic rate of glucose consumption in different regions of brain [22] along with regional cerebral blood flow in occipital cortex and cerebellum [23]. Animal studies have revealed an increase in local cerebral glucose utilization [24], cerebral blood flow and oxygen consumption during acute nicotine administration [25]. Acute and chronic nicotine exposure increased the expression of enzymes involved in glycolysis and Kreb’s cycle [26]. However, the implication of increased enzymatic activity upon nicotine exposure on the cerebral metabolism remains to be evaluated. Most of these studies have investigated the acute effects of nicotine on cerebral metabolism in human and rats. The effects of chronic nicotine on neuronal (glutamatergic and GABAergic) and astroglial metabolism in mice and rats are poorly understood.

Exposure to nicotine leads to activation, desensitization and up-regulation of nAChRs [27]. A single exposure to nicotine has been shown to transiently increase GABAergic transmission, which is followed by a persistent depression of these inhibitory inputs due to desensitization of nAChRs [6], [28], [29]. Simultaneously, nicotine enhances the glutamatergic transmission through nAChRs that desensitize less than that of GABA neurons [30]. We hypothesize that chronic exposure to nicotine will enhance the glutamatergic, excitatory neurotransmission. In the current study, we have used an approach of co-infusion of [U-^13^C_6_]glucose and [2-^13^C]acetate along with ^1^H-[^13^C]-NMR and ^13^C-[^1^H]-NMR spectroscopy to investigate the effects of chronic nicotine exposure on neuronal and astroglial metabolism in mice. Difference in isotopomers of amino acids from [U-^13^C_6_]glucose and [2-^13^C]acetate has been utilized to measure neuronal and astroglial metabolism simultaneously. ^13^C Labeling of amino acids from glucose and acetate were used to evaluate the neuronal and astroglial glucose oxidation and neurotransmitter cycle. Our findings indicate that chronic nicotine treatment led to an increase in glucose oxidation and neurotransmitter cycling associated with glutamatergic neurons. The preliminary findings of this study have been presented at the Annual Meeting of the International Society for Magnetic Resonance in Medicine, Stockholm [31].

## Materials and Methods

### Animal Preparation

All animal experiments were performed under protocols approved by the Institutional Animal Ethics Committee of the Centre for Cellular and Molecular Biology,Hyderabad, India. One month old male C57BL/6 mice were used for the study. Mice were maintained at 22°C and 60% relative humidity with a 12/12 h light and dark cycle and received standard chow and water *ad libitum*. Mice were divided into three groups: Group (i) mice treated with normal saline (0.9% NaCl) (n = 6), Group (ii) mice treated with 0.5 mg/kg nicotine (n = 6, sc), Group (iii) mice treated with 2.0 mg/kg nicotine (n = 6, sc). Nicotine dose was selected based on previous studies where nicotine, 0.1 to 4 mg/kg, has been used in different experimental paradigms [25], [26], [32], [33], [34], [35], [36]. Mice in Group (ii) and (iii) received nicotine hydrogen tartrate (SIGMA, 0.1 ml, sc) at a dose of 1.4 and 5.7 mg/kg body weight, which is equivalent to 0.5 and 2 mg/kg nicotine free base, 3 times a day every 8 h for 4 weeks. The control mice received 0.1 ml normal saline (0.9% NaCl, sc) for the same period. Metabolic measurements were carried out 2 days after the last treatment to minimize post traumatic shock with the nicotine injection.

### Infusion of [U-^13^C_6_]Glucose and [2-^13^C]Acetate

For metabolic measurements, overnight (12–14 h) fasted mice were anesthetized with urethane (1.5 g/kg, ip) and the tail vein was cannulated for the infusion of ^13^C labeled substrates. Body temperature was maintained around 37°C using a heating pad connected to a temperature-regulated circulating water bath. The respiration rate was monitored using a Biopack device. Solution of [U-^13^C_6_]glucose (0.225 mol/L) and [2-^13^C]acetate (0.8 mol/L) (99 atom %; Cambridge Isotopes, Andover, MA, USA) was administered 45 min after injection of urethane, using a bolus-variable rate infusion for 20 min as described previously [37], [38]. Blood was collected from the retro-orbital sinus artery by using a fine capillary during the last minute of the infusion. Plasma was obtained by centrifugation and stored at −80°C. At the end of the infusion, the anesthetized mice head were frozen *in situ* using liquid nitrogen [37], [39].

### Extraction of Metabolites from Brain Tissue

Brain was dissected in cryostat maintained at −20°C to isolate cortex, subcortex (hippocampus, striatum, thalamus and sub-thalamus regions of brain), cerebellum and olfactory bulb (OB), and stored at −80°C till further analysis. Metabolites were extracted from frozen brain tissues as described previously [40]. Tissues were powdered with 0.1 mol/L HCl (1∶2 v/w) in methanol using a glass homogenizer maintained in a dry-ice-ethanol bath. Following transfer to a wet ice-bath, a known quantity of [2-^13^C]glycine (0.2 µmol) was added as an internal concentration reference. The suspension was homogenized with phosphate buffered ice-cold ethanol (90%) until no visible pieces of tissue remained. The homogenate was centrifuged at 14,000 g for 30 min at 4°C. The supernatant was passed through a chelex-100 resin column (200–400 mesh, Bio-Rad Laboratories, Hercules, CA, USA), eluted with de-ionized water and lyophilized. The dried powder was dissolved in buffered (phosphate = 25 mmol/L, pH = 7) deuterium oxide containing 0.25 mmol/L sodium 3-trimethylsilyl [2, 2, 3, 3-D4]-propionate (TSP) for NMR analysis.

### Analysis of Plasma Glucose and Acetate Enrichment

Plasma (100 µl) was mixed with deuterium oxide (450 µl) containing TSP (0.25 mmol/L), and passed through a centrifugal filter (10-kDa cut off) to remove macromolecules prior to NMR analysis. Isotopic ^13^C enrichment of glucose and acetate was determined in the plasma using ^1^H NMR spectroscopy at 600 MHz (Bruker AVANCE II) spectrometer. The percent ^13^C enrichment of glucose-C1α and acetate-C2 was calculated by dividing the intensity of the two ^13^C satellites by the total (^12^C+^13^C) intensity at 5.23 and 1.91 ppm, respectively.

### NMR Analysis of Brain Tissue Extract

^1^H-[^13^C]-NMR spectra of the brain tissue extracts were acquired at 600 MHz NMR spectrometer (Bruker AVANCE) [13], [37]. Concentration of metabolites was determined relative to [2-^13^C]glycine. The ^13^C enrichment of cerebral metabolites was calculated from the ratio of the area in the ^1^H-[^13^C]-NMR difference (^13^C-labeled spectra) spectrum to that of the non-edited (^12^C+^13^C) spectrum. In addition, ^1^H decoupled ^13^C NMR (^13^C-[^1^H]-NMR) spectra of the brain tissue extracts were also acquired at 150 MHz. The isotopomer Intensity of Glu_4_, GABA_2_ and Gln_4_ was determined by using a peak fitting method provided in Topspin software (Bruker Biospin).

### Metabolism of [U-^13^C_6_]Glucose and [2-^13^C]Acetate in the Brain

**Figure 1:**
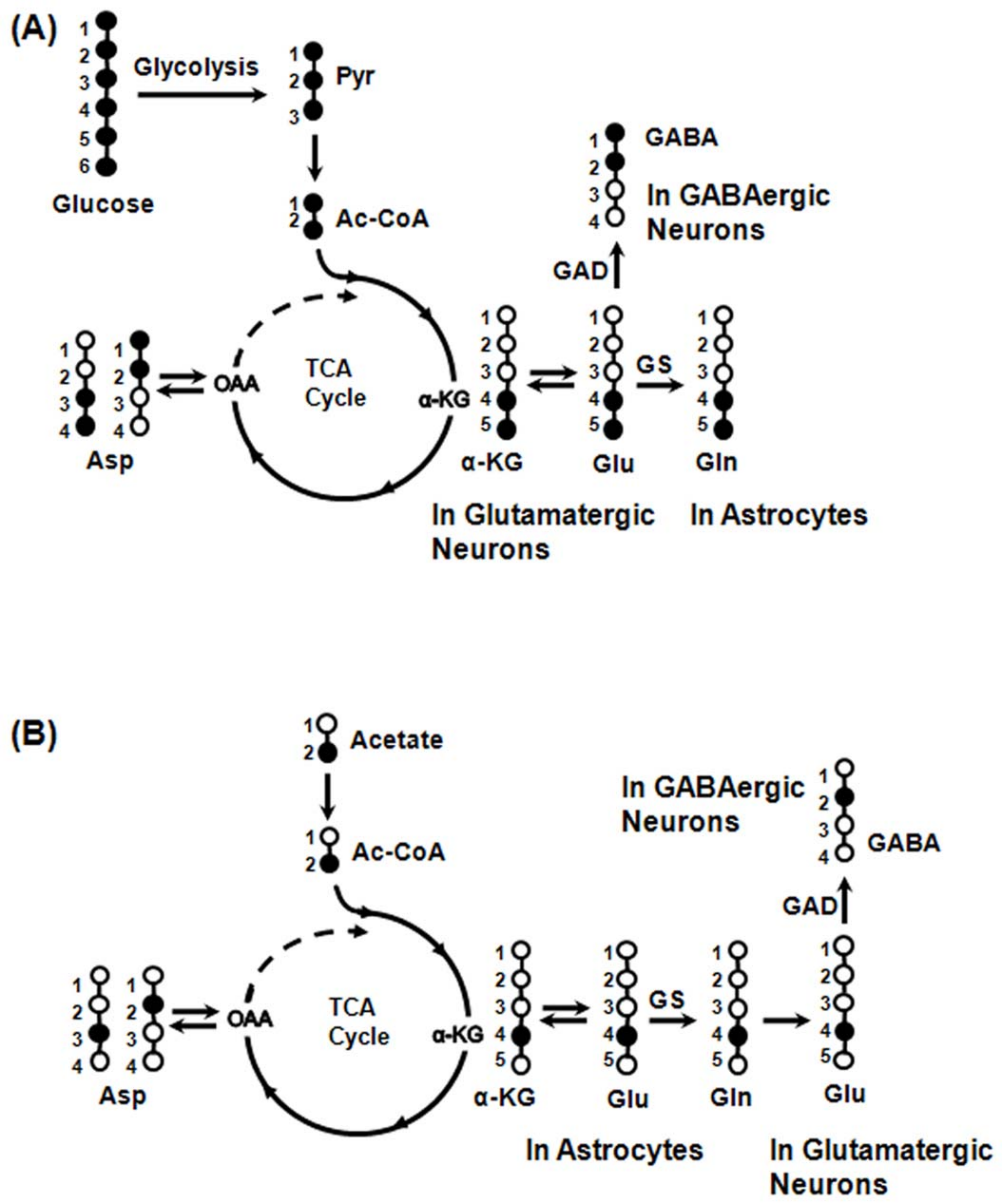
Depiction of ^13^C labeling of cerebral metabolites from labeled glucose and acetate. (**A**) Metabolism of [U-^13^C_6_]glucose via glutamatergic and GABAergic TCA cycles labels Glu_4,5_ which is de-carboxylated to GABA_1,2_ in GABAergic neurons by GAD. Labeling of Gln_4,5_ occurs from Glu_4,5_ and GABA_1,2_ via glutamate-glutamine and GABA-glutamine cycle. (**B**) As transporters of acetate are present on astroglia only, [2-^13^C]acetate is selectively transported and metabolized in these cells and labels Gln_4_ by the combined action of astroglial TCA cycle and glutamine synthetase. Neurotransmitters Glu_4_ and GABA_2_ are labeled from Gln_4_ via glutamate-glutamine and GABA-glutamine substrates cycling between astroglia and neurons. Isotopomers of Glu_4_, GABA_2_ and Gln_4_ derived from [U-^13^C_6_]glucose and [2-^13^C]acetate are different. Open and filled circles represent ^12^C and ^13^C atoms, respectively.

Metabolism of labeled glucose and acetate in the brain is depicted in Figure 1. While [U-^13^C_6_]glucose is mostly metabolized by neurons, [2-^13^C]acetate is selectively transported to astroglia [41] and gets metabolized there. Metabolism of [U-^13^C_6_]glucose via glutamatergic and GABAergic TCA cycles labels Glu_4,5_ which is de-carboxylated to GABA_1,2_ in GABAergic neurons [42]. Release of Glu_4,5_ and GABA_1,2_ from the respective neurons, followed by uptake and metabolism in astroglia via glutamate-glutamine and GABA-glutamine cycling pathways labels Gln_4,5_. Further metabolism via TCA cycle leads to labeling of [3-^13^C]Glu/Gln/GABA, [4-^13^C]GABA, [1,2,-^13^C_2_]Glu/Gln, [3,4,5-^13^C_3_]Glu/Gln, [1,2,4,5-^13^C_4_]Glu/Gln, [1,2,3-^13^C_3_]GABA and [1,2,4-^13^C_3_]GABA. [U-^13^C_6_]Glucose metabolism in astrocytes via pyruvate carboxylase pathway incorporates label into Gln_2,3_ and Glu_2,3_ (GABA_3,4_).

[2-^13^C]Acetate metabolism in astrocyte labels Gln_4_ by a combined action of astrocytic TCA cycle and glutamine synthetase [43]. Neuronal Glu_4_ and GABA_2_ are labeled from Gln_4_ via glutamate-glutamine and GABA-glutamine substrates cycling between astroglia and neurons, respectively. Further metabolism in TCA cycle incorporates label into [2-^13^C]Glu/Gln, [4-^13^C]GABA, [3-^13^C]Glu/Gln/GABA, [3,4-^13^C_2_]Glu/Gln, [2,4-^13^C_2_]Glu/Gln/GABA and [2,3-^13^C_2_]GABA. Isotopomers of amino acids derived from [U-^13^C_6_]glucose and [2-^13^C]acetate via the first turn of TCA cycle are distinct. However, the isotopic differences in the labeling of amino acids from [U-^13^C_6_]glucose and [2-^13^C]acetate are lost upon their further metabolism by TCA cycle.

Changes in labeling of Glu_4,5_, GABA_1,2_ and Gln_4,5_ from [U-^13^C_6_]glucose will indicate an alteration in glutamatergic, GABAergic TCA cycle and total neurotransmitter cycle fluxes, respectively upon chronic nicotine exposure. Similarly, an alteration in the labeling of Glu_4_, GABA_2_ and Gln_4_ from [2-^13^C]acetate will signify changes in the rate of glutamate-glutamine, GABA-glutamine neurotransmitter cycle and oxidation of acetate by astroglia with nicotine treatment.

### Contribution of Glucose and Acetate for Amino Acids Labeling

The fractional contribution of glucose and acetate for the labeling of Glu-C4 was determined from the isotopomer as following:
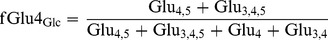
(1)

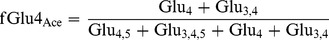
(2)where Glu_4,5_, Glu_3,4,5_, Glu_4_ and Glu_3,4_ represent intensity of [4,5-^13^C_2_]-, [3,4,5-^13^C_3_]-, [4-^13^C]- and [3,4-^13^C_2_]glutamate, respectively, in ^13^C NMR spectrum.

Similarly, the contribution of glucose and acetate to GABA-C2 and Gln-C4 labeling was also determined. ^13^C Labeling of amino acids from [U-^13^C_6_]glucose and [2-^13^C]acetate was calculated by multiplying the percent enrichment of the amino acids (obtained from the ^1^H-[^13^C]-NMR spectrum) with the fractional contribution of the corresponding substrates (obtained using Eq. 1 or 2). In addition to [2-^13^C]acetate, Glu_4_/Gln_4_/GABA_2_ resonance are also contributed by ^13^C natural abundance (1.1%). Hence, the contribution of [2-^13^C]acetate to amino acids labeling (Glu-C4, GABA-C2 and Gln-C4) was corrected for the natural abundance by subtracting 1.1% from the deconvolved ^13^C labeling. The deconvolved enrichment of amino acids from [U-^13^C_6_]glucose and [2-^13^C]acetate was normalized with corresponding ^13^C labeling of precursors in plasma. The normalized enrichment of amino acids was multiplied to the corresponding concentration to determine concentration of ^13^C labeled amino acids during 20 min of infusion.

### Statistical Analysis

Statistical analysis was carried out by using the Data Analysis Tool package of Microsoft Excel 2007. Two factor ANOVA was performed to determine the global difference in the concentration and labeling of amino acids between nicotine-treated and control mice within the same region of the brain. To correct for the multiple comparisons incurred by different metabolites and ^13^C concentrations, the α value was set to 0.005 and 0.0083, respectively so as to minimize the possibility of Type I errors. Further, differences at individual amino acid level were evaluated by a single factor ANOVA. All results are presented as mean ± SEM.

## Results

### Behavior and Physiology with Nicotine Treatment

Mice appeared immobile for 10 to 15 min after nicotine administration in the first couple of days of the treatment. The time of immobilization reduced to zero after a week of the treatment. There was no symptom of seizures/convulsions even at a dose of 2.0 mg/kg. Nicotine-treated animals appeared hyperactive during the latter half of the treatment. There was a mild reduction in the weight of mice during the first week of nicotine treatment which regained the weight in the latter part of the treatment such that there was not much difference in the weight of control and nicotine treated mice after 4 weeks. The respiration rate (Control: 241±18 breaths/min; Nicotine (0.5 mg/kg): 257±11 breaths/min; Nicotine (2.0 mg/kg): 246±11 breaths/min) during [U-^13^C_6_]glucose and [2-^13^C]acetate infusion (under urethane anesthesia) was similar (p>0.1) in nicotine-treated and control mice. Plasma urethane level was not significantly different (p>0.13, single Factor ANOVA) among control (32.4±3.2 mM, n = 5) and nicotine-treated (nicotine 0.5 mg/kg, 28.6±1.8 mM, (n = 6); nicotine 2.0 mg/kg, 26.6±2.4 mM, n = 6) mice, suggesting that chronic nicotine treatment did not alter the metabolism of urethane.

### Plasma Glucose and Acetate Enrichment

The ^13^C enrichment of glucose and acetate was measured using ^1^H NMR spectrum of plasma obtained at the last minute of [U-^13^C_6_]glucose and [2-^13^C]acetate infusion. ^13^C Labeling of glucose-C1 and acetate-C2 in control mice were 33.5±1.3% and 88.1±0.7%, respectively (Table 1). There was no significant (p = 0.11) difference in the plasma labeling of [U-^13^C_6_]glucose and [2-^13^C]acetate following chronic nicotine treatment.

**Table 1:** ^13^C Enrichment of glucose and acetate, and concentration of urethane in plasma of control and nicotine treated mice.

Substrate	Control	Nicotine	
	Saline (n = 6)	0.5 mg/kg(n = 6)	2 mg/kg(n = 6)
[1-^13^C]Glucose (%)	33.5±1.3	39.4±2.3	30.7±1.6
[2-^13^C]Acetate (%)	88.1±0.7	85.0±1.0	87.7±0.6
Urethane (mM)	32.4±3.2	28.6±1.8	26.4±2.4

Percent ^13^C enrichment of [1]-[^13^]glucose and [2-^13^C]acetate was measured from resonances at 5.2 and 1.9 ppm, respectively, in ^1^H NMR spectrum of plasma. Concentration of urethane was estimated from triplet resonance at 1.24 ppm relative to formate. Values represent mean±SEM. p = 0.11.

**Figure 2:**
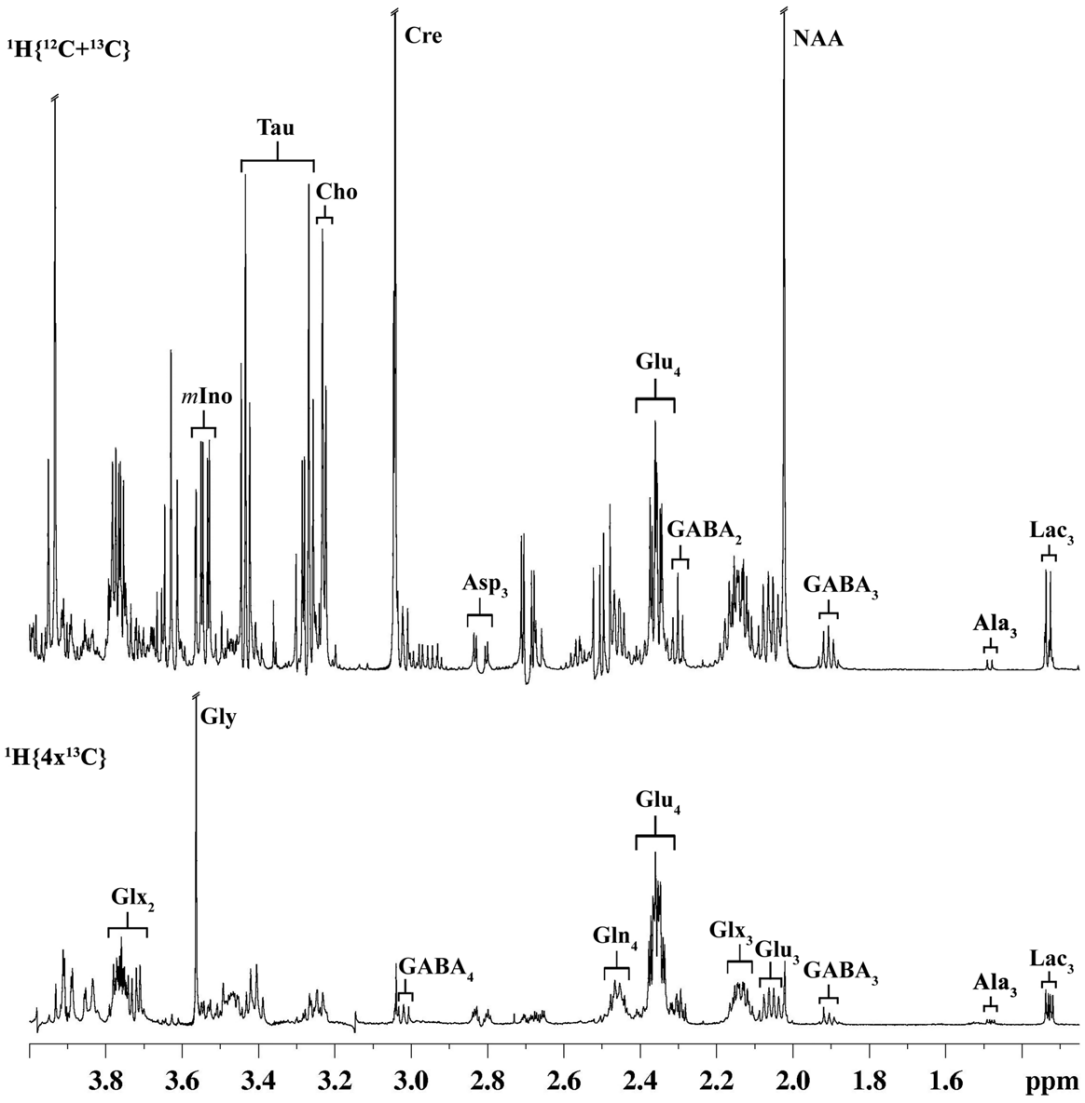
Representative ^1^H-[^13^C]-NMR spectrum of cortical tissue extract of nicotine-treated mouse. The upper spectrum (^1^H{^12^C+^13^C}) depicts total concentration of metabolites, while the lower spectrum (^1^H{4×^13^C}) represents the ^13^C labeled metabolites. Peak labels are: Ala_3_, alanine-C3; GABA, γ-amino butyric acid; Gln_4_, glutamine-C4; Glu_4_, glutamate-C4; Glu_3_, 0.5 × glutamate-C3; Glx_3,_ (0.5 × glutamate + glutamine)-C3; Asp_3_, aspartate-C3; Cre, creatine; NAA, N-acetyl aspartate; Cho, choline, m-Ino, myo-inositol; Tau, taurine.

### Effect of Chronic Nicotine Treatment on Cerebral Metabolism

Cerebral metabolism was investigated by monitoring ^13^C labeling of brain metabolites using ^1^H-[^13^C]-NMR spectrum (Figure 2). Labeling of glutamate, glutamine, GABA and aspartate could be seen at different carbon positions (lower spectrum). Chronic nicotine (0.5 mg/kg) treatment led to a significant (F[1], [10] = 13.0, p = 0.0048, n = 6,6) increase in the percent enrichment of cortical GABA_2_ when compared with control, while no change was seen in the labeling of other amino acids (Table 2). An increase in the Glu_3_/Glu_4_ ratio from 0.35±0.01 for control to 0.42±0.01 for nicotine (F[1], [10] = 14.18, p = 0.0037, n = 6,6) indicates increase in cortical metabolism [37] in mice treated with nicotine (0.5 mg/kg). Nicotine (2.0 mg/kg) treatment resulted in an increase (*p*<0.01) in the labeling of all cortical metabolites except Gln_4_. Moreover, the Glu_3_/Glu_4_ ratio was also increased from 0.35±0.01 for control to 0.43±0.01 for nicotine (F[1], [10] = 23.3, p = 0.0007, n = 6,6), indicating that an increase in the rate of glutamatergic TCA cycle in cortex of mice exposed to 2.0 mg/kg nicotine. Increase in rate of glutamatergic TCA cycle with chronic nicotine (2.0 mg/kg) treatment was also observed in the cerebellum and sub-cortical regions (Table 2). Olfactory bulb showed a different activation pattern upon nicotine treatment. Nicotine exposure (0.5 mg/kg) increased the labeling of GABA_3_ (F[1], [10] = 6.37, p = 0.03, n = 6,6) with no significant (*p*>0.2) change in the labeling of other amino acids. Exposure to 2.0 mg/kg nicotine increased (*p*<0.02) percent labeling of Glu_3_, GABA_2_ and GABA_3_. In summary, glutamatergic metabolism was increased in the cortex, subcortex, cerebellum and OB with chronic nicotine exposure (Table 2).

**Table 2:** ^13^C Enrichment of cerebral amino acids from [U-^13^C_6_]glucose and [2-^13^C]acetate in different brain regions.

Brain Regions	Treatment Groups	^13^C Enrichment (%)						
		Glu_4_	GABA_2_	Gln_4_	Glu_3_	GABA_3_	Glu_3_/Glu_4_	GABA_3_/GABA_2_
Cortex	Control (n = 6)	29.8±1.2	17.5±0.5	23.5±1.5	10.6±0.8	9.0±0.5	0.35±0.01	0.51±0.02
	Nic (0.5 mg, n = 6)	31.0±1.0	20.7±0.7**	24.2±1.5	13.1±0.5	11.0±0.5	0.42±0.01**	0.53±0.01
	Nic (2.0 mg, n = 6)	34.8±1.0**	22.1±0.6**	27.7±1.6	14.9±0.7**	12.2±0.5**	0.43±0.01**	0.55±0.02
Subcortex	Control (n = 6)	30.5±1.3	16.6±0.8	27.2±1.3	10.8±0.8	8.0±0.5	0.35±0.01	0.48±0.01
	Nic (0.5 mg, n = 6)	31.5±0.9	18.5±0.4	26.6±1.7	13.3±0.4*	9.5±0.2*	0.42±0.01**	0.51±0.01
	Nic (2.0 mg, n = 6)	35.8±1.2*	20.5±0.8**	33.2±1.8*	15.4±0.5**	10.8±0.5**	0.43±0.01**	0.53±0.01*
Cerebellum	Control (n = 6)	27.3±1.1	20.9±1.1	22.7±1.5	9.2±0.7	10.3±1.0	0.34±0.01	0.49±0.02
	Nic (0.5 mg, n = 6)	29.4±1.0	24.3±0.8*	23.1±1.3	12.0±0.5*	14.2±0.9*	0.41±0.01**	0.58±0.02*
	Nic (2.0 mg, n = 6)	32.5±1.0**	26.5±1.2**	28.0±1.3*	12.8±0.6**	15.1±0.7**	0.39±0.01**	0.57±0.01**
Olfactory Bulb	Control (n = 6)	36.6±1.5	13.9±0.5	28.7±1.8	14.9±1.0	6.5±0.4	0.41±0.02	0.46±0.01
	Nic (0.5 mg, n = 6)	36.1±1.9	14.8±0.5	26.9±1.9	16.3±0.8	8.4±0.4*	0.45±0.01*	0.54±0.02**
	Nic (2.0 mg, n = 6)	42.0±3.1	17.5±0.8**	34.4±2.1	19.7±1.4*	9.1±0.5**	0.47±0.01**	0.52±0.01**

Percent ^13^C enrichment was measured in brain tissue extract using ^1^H-[^13^C]-NMR spectroscopy. Values represent mean±SEM.

* *p*<0.05,

** *p*<0.01 indicate significance of differences when compared to respective control.

### Effect of Chronic Nicotine on Glutamatergic and GABAergic Pathways

**Figure 3:**
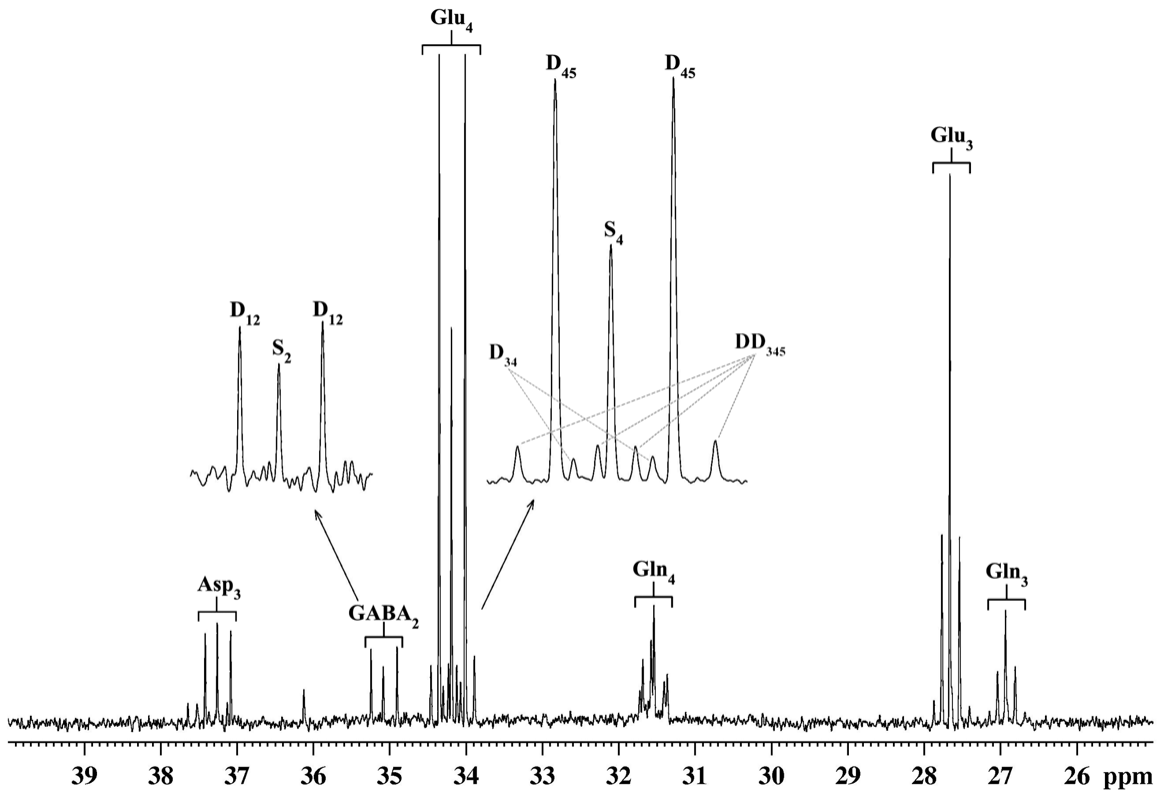
Representative ^13^C-[^1^H]-NMR spectrum of cortical tissue extract of nicotine-treated mouse. The inset depicts the resonances for Glu_4_ and GABA_2_. In Glu_4_, D_45_ and DD_345_ represent [4,5-^13^C_2_]glutamate and [3,4,5-^13^C_3_]glutamate, and are the product of glucose metabolism in the first and second turn of the TCA cycle, respectively. S_4_ and D_34_ indicate [4-^13^C]glutamate and [3,4-^13^C_2_]glutamate which are labeled from [4-^13^C]glutamine and [3,4-^13^C_2_]glutamine, respectively. [4-^13^C]Glutamine and [3,4-^13^C_2_]glutamine are the products of [2-^13^C]acetate metabolism in the first and second turn of the astrocytic TCA cycle, respectively. In GABA_2_, D_1,2_ represents [1,2-^13^C_2_]GABA which is the product of direct metabolism of [U-^13^C_6_]glucose in the GABAergic TCA cycle, while S_2_ ([2-^13^C]GABA) is the result of the labeling from [2-^13^C]acetate via astrocytic TCA cycle followed by trafficking into GABAergic neurons.

**Figure 4:**
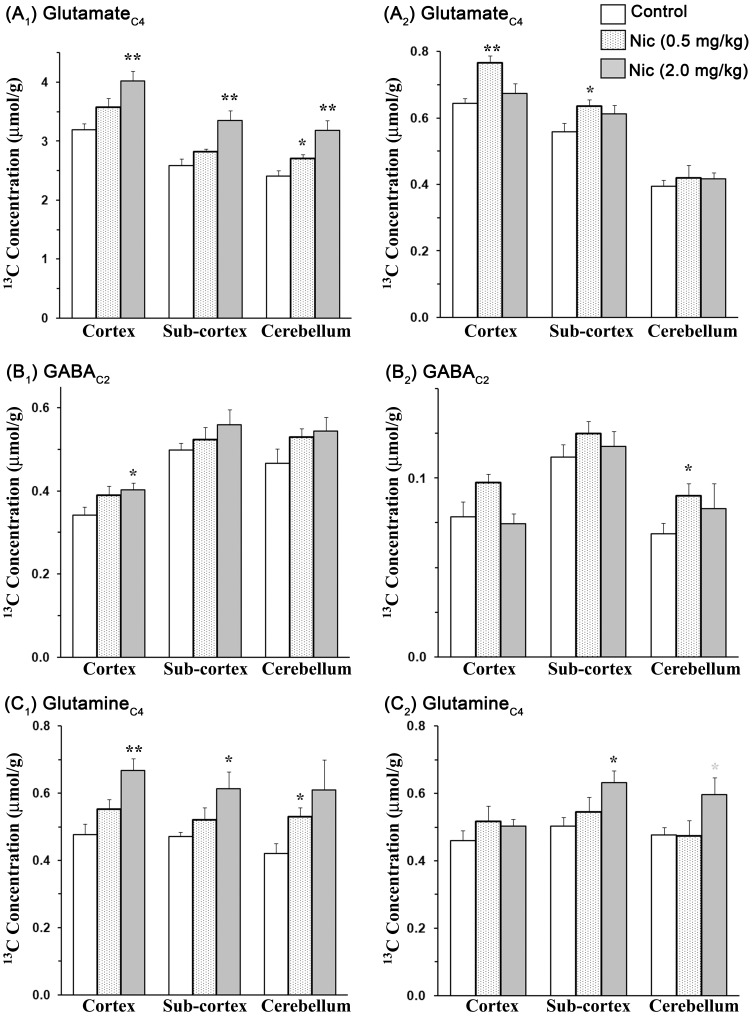
Concentration of ^13^C labeled (A) glutamate-C4, (B) GABA-C2 and (C) glutamine-C4 from [U-^13^C_6_]glucose and [2-^13^C]acetate. The subscripts 1 and 2 with alphabet A, B and C represent labeling from [U-^13^C_6_]glucose and [2-^13^C]acetate, respectively.^ 1^H-[^13^C]-NMR measured ^13^C enrichment was deconvolved for the contribution of [U-^13^C_6_]glucose and [2-^13^C]acetate using Eq. 1 and 2, respectively. ^13^C Concentrations of amino acids were obtained by multiplying the normalized deconvolved labeling with the total concentration. Values represent mean±SEM. *p<0.05, **p<0.01 indicate significance of differences when compared to the respective controls.

Isotopomers of the amino acids were measured from the ^13^C-[^1^H]-NMR spectrum (Figure 3). The ^1^H-[^13^C]-NMR measured enrichment of Glu_C4_ was de-convolved for its contribution from [U-^13^C_6_]glucose and [2-^13^C]acetate using Eq. 1 and 2, respectively (Table 3). ^13^C Concentration of labeled amino acids from [U-^13^C_6_]glucose and [2-^13^C]acetate in nicotine- and normal saline (0.9% NaCl)-treated mice is presented in Figure 4. [4-^13^C]Glutamate labeling from [U-^13^C_6_]glucose was increased significantly (p<0.05) in the cortical, subcortical and cerebellum regions in mice exposed to nicotine (2.0 mg/kg). In addition, [4-^13^C]glutamine labeling from [U-^13^C_6_]glucose was also enhanced significantly (p<0.02, n = 6,6) in the cortex and subcortex upon nicotine treatment (2.0 mg/kg). Furthermore, [4-^13^C]glutamate labeling from [2-^13^C]acetate was found to be increased significantly (p<0.02, n = 6,6) in the cortical (Control 0.64±0.02 µmol/g; Nicotine 0.76±0.02 µmol/g) and subcortical (Control 0.56±0.03 µmol/g; Nicotine 0.63±0.02 µmol/g) regions of the brain in mice treated with 0.5 mg/kg nicotine (Figure 4, Table S1). Although there was a significant increase (p<0.05) in the percent ^13^C labeling of GABA_2_ with nicotine treatment in different regions of the brain (Table 2 and 3), the total level of GABA was reduced slightly with chronic nicotine exposure (data not shown). As a result, the concentration of newly synthesized [2-^13^C]GABA (total concentration×^13^C enrichment/100) was similar among different treatment groups in various regions of the brain except an elevation of [2-^13^C]GABA with nicotine treatment (2.0 mg/kg) (p<0.05) in the cortical region. Furthermore, concentration of labeled glutamine-C4 from [2-^13^C]acetate was increased (p<0.05) in the subcortex and cerebellum regions of the brain with chronic nicotine exposure, suggesting that astroglial activity is enhanced in these brain regions (Table S1). In summary, increased [4-^13^C]glutamate labeling from glucose and acetate with chronic nicotine treatment indicates an increase in glucose oxidation by glutamatergic neurons in all regions of the brain and glutamate-glutamine neurotransmitter cycle in the cortical and subcortical regions. The finding of increased [4-^13^C]glutamine from [U-^13^C_6_]glucose in nicotine treated mice further corroborates with the increased glutamate-glutamine cycle with chronic nicotine exposure. GABAergic and astroglial function are found to enhanced only in selected brain regions.

**Table 3:** Deconvolved ^13^C labeling of cerebral amino acids from [U-^13^C_6_]glucose and [2-^13^C]acetate in different brain regions.

Brain Region	Treatment	GluC4		GABAC2		GlnC4	
		Glucose	Acetate	Glucose	Acetate	Glucose	Acetate
Cortex	Control (n = 6)	22.2±1.1	4.5±0.1	12.2±0.3	2.8±0.2	9.6±0.6	9.2±0.5
	Nic (0.5 mg) (n = 6)	23.9±0.7	5.2±0.2*	15.1±0.5**	3.8±0.2*	11.1±0.4	10.5±1.0
	Nic (2.0 mg) (n = 6)	26.6±0.8**	4.5±0.2	16.0±0.6**	3.0±0.2	12.3±0.7*	9.3±0.4
Subcortex	Control (n = 6)	22.5±1.0	4.8±0.1	11.6±0.6	2.6±0.1	10.6±0.5	11.2±0.5
	Nic (0.5 mg) (n = 6)	24.2±0.5	5.4±0.2*	13.6±0.3*	3.2±0.1**	11.6±0.8	12.2±1.0
	Nic (2.0 mg) (n = 6)	26.9±0.8**	4.9±0.1	14.3±0.6**	3.0±0.1*	12.6±0.8	13.0±0.2*
Cerebellum	Control (n = 6)	21.1±1.0	3.5±0.2	16.1±0.9	2.4±0.2	8.4±0.7	9.5±0.4
	Nic (0.5 mg) (n = 6)	24.0±0.9	3.7±0.3	19.3±0.7*	3.3±0.3*	11.0±0.8*	9.6±0.6
	Nic (2.0 mg) (n = 6)	25.7±0.8**	3.4±0.2	20.2±1.1*	3.1±0.5	10.8±1.4	10.7±0.8

The ^1^H-[^13^C]-NMR measured percent labeling was deconvolved for [U-^13^C_6_]glucose and [2-^13^C]acetate by using the isotopomer information obtained in ^13^C-[^13^H]-NMR and normalized with the percent ^13^C labeling of precursors (glucose and acetate) in plasma. Values represent mean±SEM.

* *p*<0.05,

** *p*<0.01 indicate significance of differences when compared to respective control.

## Discussion

Although the acute effects of nicotine on cerebral blood flow and glucose utilization have been investigated in rats and human brain, there is scarcity of data pertaining to the chronic effect of nicotine on cerebral function. To the best of our knowledge, this is the first study which has evaluated the effect of chronic nicotine on glutamatergic, GABAergic and astroglial glucose oxidation and neurotransmitter cycle in different regions of the mouse brain. Using an approach of co-infusion of [U-^13^C_6_]glucose and [2-^13^C]acetate together with ^13^C NMR spectroscopy, we found that chronic treatment of nicotine increased excitatory activity in all regions of the brain, while inhibitory function was enhanced in the cerebral cortex only.

### Nicotine and Cerebral Glucose Utilization

The effects of acute nicotine treatment on cerebral metabolism have been studied in rats and human brain. Autoradiographic investigations have shown increased utilization of glucose in rats treated with nicotine ranging from 0.1 to 1.75 mg/kg [34]. The highest stimulation in the most brain areas was obtained with 0.3 mg/kg nicotine. Intravenous injection of nicotine (4 mg/kg, every 30 min) has been shown to increase cortical glucose oxidation and cerebral blood flow in rats anesthetized with morphine [25]. Effect of nicotine on regional cerebral glucose metabolism and blood flow has been investigated by nasal spray of nicotine in humans [23]. Increase in cerebral glucose consumption was reported in the cerebral left inferior frontal gyrus, left posterior cingulate gyrus and right thalamus [22]. Positron emission tomography study have suggested an increase in blood flow in the visual cortex and cerebellum, and a decrease in the right hippocampus and ventral striatum including the nucleus accumbens in overnight abstinent smokers followed by nasal spray [44]. However, a very recent study conducted in awake rats indicated a significant reduction in neuronal glucose oxidation and neurotransmitter cycle flux with 0.7 mg/kg nicotine exposure [45]. The effects of nicotine on the activity of various glycolytic and TCA cycle enzymes have been studied under acute and chronic nicotine treatments in frontoparietal regions and subcortical nuclei of the rat brain [26]. Both acute as well as chronic exposure of nicotine increases enzymatic activities in the frontoparietal cortex but deeper layers of the cortex, substantia nigra, caudate-putamen, nucleus accumbens or nucleus basalis magnocellularis did not exhibit increase in activity as high as seen in the frontoparietal cortex. It is noteworthy that most of these studies have investigated effects of acute nicotine exposure on brain energy metabolism and the consequences of chronic nicotine exposure on glutamatergic, GABAergic and astroglial function is still an open problem. Furthermore, with the exception to findings of Wang et al [45], most of the studies conducted in rats and human have indicated a stimulatory role for nicotine. Our findings of increased energy metabolism associated with glutamatergic neurons in the cortex, subcortex and cerebellum in mice under chronic nicotine exposure is consistent with increased glucose consumption observed under acute nicotine in autoradiography [34], PET [44] and NMR [25] studies as well as with reported increase in enzymatic activity under acute and chronic nicotine exposure [26].

### Effect of Chronic Nicotine Treatment on Glutamatergic and GABAergic Activity

Nicotine exposure leads to activation, desensitization and up-regulation of nAChRs [27]. A single nicotine exposure increases dopamine levels in the mesolimbic reward system for hours. Nicotine exposure has been shown to increase GABAergic transmission transiently [6], [29], which is followed by a persistent depression of these inhibitory inputs due to desensitization of nAChRs. Simultaneously, nicotine enhances the glutamatergic transmission through activation of nAChRs that desensitizes lesser than GABAergic neurons [28], [30]. The net effect of these modulatory changes is in a shift towards greater excitability of dopaminergic neurons in ventral tegmental area (VTA). Thus, both activation and desensitization of nAChRs contribute to nicotine’s effect on the excitability of dopaminergic neurons [46]. Although, activation and desensitization of GABAergic neurons has been reported under acute condition, desensitization of GABAergic neurons might also be expected with chronic nicotine treatment. The data from the current study suggest an increase in glucose oxidation and neurotransmitter cycling by glutamatergic neurons in the cortex, subcortex and cerebellum upon chronic nicotine exposure. However, GABAergic metabolism was found to increase in the cortex and remain unperturbed in the subcortical and cerebellum regions. It may be possible that local increase in GABAergic metabolism in the subcortex and cerebellum upon chronic nicotine exposure might have been nullified due to measurement carried out in macroscopic brain volume.

The observations of high rate of glutamine labeling from [1-^13^C]glucose in *in vivo* ^13^C NMR studies indicate that glutamine is synthesized primarily from released neuronal glutamate [12], [47]. These studies have further established that the neurotransmitter cycle comprising of glutamate-glutamine accounts for major fraction (>80%) of glutamine synthesis [12], [15], [48]; importantly, it has a metabolic rate similar to neuronal glucose oxidation [16], [17]. High rate of the neurotransmitter cycle has been found in NMR studies of human cerebral cortex [49], [50], [51]. ^13^C NMR studies have further established that the rate of neurotransmitter cycle and neuronal mitochondrial glucose oxidation increased proportionately with a near 1∶1 slope [8], [16], [17], indicating that neurotransmitter energetics is supported by neuronal oxidative glucose metabolism. Therefore, increased glucose oxidation by glutamatergic and GABAergic neurons could be ascribed to increased glutamatergic and GABAergic neurotransmission upon chronic nicotine exposure.

### Relevance to Nicotine Addiction

A thorough understanding of molecular processes of nicotine addiction is lacking. Nicotine addiction has been related to the up-regulation of nAChRs, desensitization and modulation of the dopaminergic and glutamatergic systems [52]. In the classical model, it is hypothesized that chronic nicotine exposure leads to up-regulation of nAChRs by increasing their half-life [53], [54]. nAChRs modulate the release of dopamine which is important for development of nicotine addiction. The up-regulation of nAChRs due to chronic nicotine exposure is followed by desensitization via a reversible reduction in response [55]. Level of nicotine present in smokers desensitizes as well as up-regulates α4β2 without having much effect on α7. There is an increased dopamine release in the nucleus accumbens and activation of dopaminergic neurons in the VTA by nicotine levels that are obtained by smoking [56]. Furthermore, chronic nicotine reduces basal extracellular dopamine levels [57]. Systemic administration of nicotine increases the activity of dopaminergic neurons in the VTA via N-methyl-D-aspartate (glutamate) receptors on dopaminergic cell bodies [58] which is mediated by α7 receptors present on the glutamatergic terminals facilitating glutamate release [30], [59], [60]. These studies point to a significant increase in glutamate release and desensitization of GABA in VTA during nicotine exposure [6]. Our findings, along with those of others [29], [61], indicate an increase in glutamatergic activity in the cortex, subcortex, cerebellum and OB, suggesting that nicotine-addiction related increase in glutamate release may not be limited to the VTA.

### Limitations of the Study

In this study we have used an approach of co-infusion of [U-^13^C_6_]glucose and [2-^13^C]acetate together with ^1^H-[^13^C] and ^13^C-[^1^H]-NMR spectroscopy to study neuronal and astroglial metabolism in mice treated with nicotine. The low sensitivity of ^13^C-[^1^H]-NMR necessitates the use of concentrated sample i.e. more tissue for the measurement, hence compromised spatial resolution. Metabolic investigations at higher spatial resolution are difficult using the co-infusion approach. In fact, due to this limitation, we could not measure contribution of glucose and acetate to the total labeling of amino acids in the olfactory bulb. As we have combined all the subcortical (striatum, hippocampus, thalamus and hypothalamus) regions for the NMR analysis, it may be possible that the regional changes in GABA metabolism due to nicotine treatment might have subsided. Metabolic investigations at higher spatial resolution can be achieved by infusing labeled glucose or acetate individually and analyzing the ^13^C labeling of amino acids by inverse detection.

There may be a change in the expression of monocabroxylate transporters in mice treated with nicotine due to changes in the food intake. Although there was a decrease in the weight of animals during the first week of nicotine treatment, mice regained the weight in the latter part of the treatment such that there was not much difference in the weight of mice in different treatment groups at the time of study (not shown). These data suggest that food intake was similar in control and nicotine-treated mice at the time of metabolic analysis. Further, NMR analysis of plasma did not show significant presence of β-hydroxy butyrate, a marker for ketone bodies. Therefore, the possibility of increase in monocarboxylate transporters and increased utilization of ketogenic substrates in nicotine-treated mice is minimal. An increase utilization of ketone bodies may reduce the ^13^C labeling of acetylCoA, the precursor for TCA cycle, which will reduce the labeling of Glu_C4_, and thus the TCA cycle flux evaluated using a single point measurement. Therefore, single point measurement with a short infusion of ^13^C labeled substrates provides qualitative information for the changes in the metabolism under chronic nicotine exposure. This approach has been utilized in many studies to gain insights in metabolism under different conditions [37], [62], [63]. The quantitative changes in metabolic rates associated with glutamatergic and GABAergic pathways could be obtained by constructing the time course of labeling of brain amino acids during infusion of labeled substrates for different time points and modeling the data by a three compartment metabolic model [15]. However, the modeling of ^13^C turnover data during co-infusion of [U-^13^C_6_]glucose and [2-^13^C]acetate is not fully understood and parameterized in different regions of brain. Hence, quantitative evaluation of the effects of chronic nicotine on metabolic rates was not attempted. The absolute changes in the value of glutamatergic and GABAergic neurotransmission and TCA cycle rates during chronic nicotine exposure is still an unanswered question, which can be addressed by modeling of the ^13^C turnover curve of amino acids from [1-^13^C]glucose as described by us earlier [15,64], and by Wang et al [45] under acute nicotine treatment. However, the directional changes in the metabolic fluxes between control and treated groups will remain the same, but the absolute values of the TCA cycle and neurotransmitter cycle may be different.

Statistical analysis of ^13^C enrichment of plasma [1-^13^C]glucose and [2-^13^C]acetate did not yield difference (p = 0.11) among different groups. Moreover, the data presented in Tables 2 & 3 and Figure 4 are normalized with the respective percent enrichment of precursors in the plasma. Hence, the differences in the ^13^C labeling of brain amino acids between different groups are due to changes in metabolism and not because of differences in the ^13^C enrichment of precursors in the plasma. Normalization with enrichment of precursors in the plasma does not rule out differential changes in the utilization of substrates used for the synthesis of acetylCoA. As mentioned above the possibility of fractional changes in the substrate utilized is minimum and will have a positive effect on metabolism.

In the present study metabolic analysis has been carried out under urethane anesthesia. It may be possible that chronic nicotine treatment stimulates liver enzymes so that urethane is metabolized with rate different in nicotine-treated mice from those in the control; therefore, the effect of anesthetic is different among the groups. However, the level of urethane in blood plasma at the time of substrates infusion was not significantly different (p>0.13, single Factor ANOVA) among control and nicotine (0.5 mg/kg and nicotine 2.0 mg/kg)-treated mice, suggesting chronic nicotine treatment did not alter urethane metabolism. Furthermore, the measured respiration rate during substrate infusion was similar (p>0.1) in control and nicotine-treated mice. Hence, anesthetic effect should be similar in both groups. As metabolic analysis was carried out 2 days after the last treatment of nicotine, the contribution of processes associated with withdrawal of nicotine on observed changes in metabolism could not be ruled out. This could be evaluated by studying the metabolism at different times after treatment of nicotine.

In conclusion, chronic nicotine exposure enhanced excitatory activity associated with glutamatergic neurons in majority of the brain regions. The inhibitory and astroglial functions were found to be perturbed in the selected brain regions following chronic nicotine treatment.

## Supporting Information

Table S1 **The concentration of ^13^C labeled amino acids was calculated by multiplying the normalized deconvolved enrichment with the total concentration.** Values represent mean±SEM. *p<0.05, **p<0.01 indicate significance of differences when compared to respective control.
(PDF)
