# Supplementary material for: Glutamatergic and GABAergic Metabolism in Mouse Brain under Chronic Nicotine Exposure: Implications for Addiction
Source: PLoS One. 2012 Jul 25;7(7):e41824. doi: 10.1371/journal.pone.0041824 (PMC3405019; doi:10.1371/journal.pone.0041824)
Supplement: Table S1 — The concentration of 13C labeled amino acids was calculated by multiplying the normalized deconvolved enrichment with the total concentration. Values represent mean±SEM. *p<0.05, **p<0.01 indicate significance of differences when compared to respective control. (PDF) [file pone.0041824.s001.pdf]

Table S1: ^{13}C Concentration of cerebral amino acids from [U-$^{13}C_6$]glucose and [2-^{13}C]acetate

Brain Region	Treatment	GluC4		GABAC2		GlnC4	
		Glucose	Acetate	Glucose	Acetate	Glucose	Acetate
Cortex	Control (n=6)	3.19±0.11	0.64±0.02	0.34±0.02	0.08±0.01	0.48±0.03	0.46±0.03
	Nic (0.5mg) (n=6)	3.57±0.16	0.76±0.02**	0.39±0.02	0.10±0.00	0.55±0.03	0.52±0.05
	Nic (2.0mg) (n=6)	4.02±0.17**	0.67±0.03	0.40±0.02*	0.07±0.01	0.67±0.04**	0.50±0.02
Subcortex	Control (n=6)	2.59±0.11	0.56±0.03	0.50±0.02	0.11±0.01	0.47±0.01	0.50±0.03
	Nic (0.5mg) (n=6)	2.82±0.06	0.63±0.02*	0.52±0.03	0.12±0.01	0.52±0.04	0.55±0.04
	Nic (2.0mg) (n=6)	3.35±0.17**	0.61±0.03	0.56±0.04	0.12±0.01	0.61±0.05*	0.63±0.04*
Cerebellum	Control (n=6)	2.41±0.09	0.39±0.02	0.47±0.04	0.07±0.01	0.42±0.03	0.48±0.02
	Nic (0.5mg) (n=6)	2.70±0.08*	0.42±0.04	0.53±0.02	0.09±0.01*	0.53±0.03*	0.47±0.05
	Nic (2.0mg) (n=6)	3.18±0.17**	0.42±0.02	0.54±0.03	0.08±0.01	0.61±0.09	0.60±0.05*

The concentration of ^{13}C labelled amino acids was calculated by multiplying the normalised deconvolved enrichment with the total concentration. Values represent mean±SEM. *$p < 0.05$, **$p < 0.01$ indicate significance of differences when compared to respective control.
